# Gamma-Irradiation Induced Functionalization of Graphene Oxide with Organosilanes

**DOI:** 10.3390/ijms20081910

**Published:** 2019-04-18

**Authors:** Kabiru Musa Aujara, Buong Woei Chieng, Nor Azowa Ibrahim, Norhazlin Zainuddin, Chantara Thevy Ratnam

**Affiliations:** 1 Department of Chemistry, Faculty of Science, Universiti Putra Malaysia, 43400 UPM Serdang, Selangor, Malaysia; kbaujara@gmail.com (K.M.A.); norhazlin@upm.edu.my (N.Z.); 2 Materials Processing and Technology Laboratory, Institute of Advanced Technology, Universiti Putra Malaysia, 43400 UPM Serdang, Selangor, Malaysia; 3 Radiation Processing Technology Division, Malaysian Nuclear Agency, 43000 Bangi, Kajang, Malaysia; chantara@nuclearmalaysia.gov.my

**Keywords:** functionalization, silanes, graphene, gamma-ray

## Abstract

Gamma-ray radiation was used as a clean and easy method for turning the physicochemical properties of graphene oxide (GO) in this study. Silane functionalized-GO were synthesized by chemically grafting 3-aminopropyltriethoxysilane (APTES) and 3-glycidyloxypropyltrimethoxysilane (GPTES) onto GO surface using gamma-ray irradiation. This established non-contact process is used to create a reductive medium which is deemed simpler, purer and less harmful compared conventional chemical reduction. The resulting functionalized-GO were characterized by Fourier transform infrared spectroscopy (FT-IR), X-ray diffraction (XRD), field emission scanning electron microscopy (FE-SEM), thermogravimetric analysis (TGA), and Raman spectroscopy. The chemical interaction of silane with the GO surface was confirmed by FT-IR. X-ray diffraction reveals the change in the crystalline phases was due to surface functionalization. Surface defects of the GO due to the introduction of silane mioties was revealed by Raman spectroscopy. Thermogravimetric analysis of the functionalized-GO exhibits a multiple peaks in the temperature range of 200–650 °C which corresponds to the degradation of chemically grafted silane on the GO surface.

## 1. Introduction

Graphene enjoys so much attention since its discovery by Andre Geim and Konstantin Novoselov in 2004 due to its unique electrical, optical, thermal, and mechanical properties which are desirable for a broad range of high tech applications in supercapacitors, batteries, flexible transparent electronic devices, composites, flexible transparent displays, and sensors [1]. Graphene is one atom thick, two-dimensional (2D) monolayer sheet of sp^2^ bonded carbon atom arranged in a hexagonal lattice. Graphene related materials that consist of chemical derivatives or structure of graphene are commonly called ‘graphene’. These include double and few layered graphene and chemically reduced graphene oxide (rGO) [2]. Potential applications of graphene is on the rise over the last decade and are nowadays covering most research field in materials science. The range of graphene chemistries, grade, and morphologies that can be processed is numerous due to the diversity of growth and surface modification mechanism which has been used on sp^3^ carbon based materials [3]. For instance, epitaxial growth, chemical vapor deposition (CVD) and mechanical cleavage of graphite leads to the formation of very high grade material with low sp^3^ carbon content [4].

The performance and quality of graphene depend on synthetic approaches and thus various techniques have been developed to produce graphene with a wide range of properties based on morphology and surface chemistry [5]. Some of the synthesis techniques of graphene includes chemical vapor deposition, exfoliation method, organic synthesis approach, solid carbon foundation, expanding of carbon nanotubes, and epitaxial process [6]. The reduction of GO fabricated from graphite through Hummer’s method is relatively cheap and fast technique to produce low grade graphene material [7]. However, large scale based processes are impractical through these methods, hence oxidation and exfoliation of graphite oxide followed by chemical reduction have been employed to prepare reduced graphene oxide sheets or chemically functionalized graphene [8]. GO is generally regarded as an oxidative state of graphene due to possession of huge amount of hydroxyl, carboxyl, and epoxide functional groups on its basal planes and edges [9]. GO is associated with higher sp^3^ and defect content across its sheets which primarily consists of residual oxygen groups and new sites generated upon exfoliation and oxidation which must be alleviated to recover the thermo-mechanical and electrical properties of pristine graphene [10].

A new frontier takes into account the surface treatment and functionalization of GO for modification of their surface with various agents in order to improve compatibility with polymers when it is used as a reinforcing agent. The oxygen containing groups available on the basal plane and edges such as hydroxyl, carboxyl, and epoxy groups provide suitable reactive sites for covalent functionalization. Due to their exceptional physical properties and their dispersion characteristics in various polymer matrices, GO and its derivatives have emerged as a new class of polymer nanocomposites as reinforcing agents [11]. 

Various functionalizing techniques have been developed for improving interfacial bonding and dispersion of GO in the polymer matrix among which silane coupling agents gained so much attention due to their bifunctional structures [12]. Coupling process can be achieved through chemical reaction between the alkoxy groups of the silane molecules and the hydroxyl groups on the graphene surface leaving the other functional groups of the silane molecule which can be amine, ethylene, epoxy, thiohydroxy, etc. unreacted [13]. These unreacted functional groups can provide reaction sites between graphene with polymer matrix thus leading to enhanced properties. Methods previously used for the functionalization of GO such as chemical methods often have their drawbacks. These methods usually involve toxic reagent, long reaction time, strict reaction conditions that limits scalability [14]. Therefore, a novel route that combines convenience of chemical synthesis and economic benefits, whilst possessing high reduction efficiency, has been an important target for scientists. 

**Figure 1:**
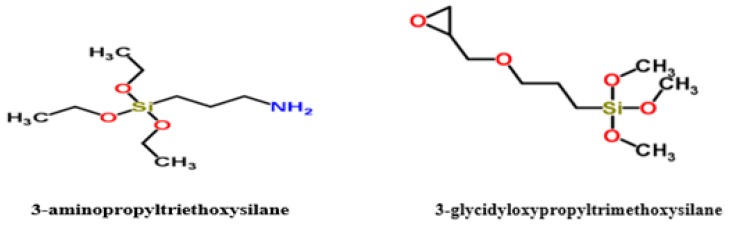
Chemical structure of 3-aminopropyltriethoxysilane (APTES) and 3-glycidylpropyltrimethoxysilane (GPTES).

Recently, scientific interest has focused on the exploitation of gamma radiation as a clean easy method for turning the physicochemical properties of GO [15]. This established non-contact process is used to create a reductive medium which is deemed simpler, purer and less harmful than conventional chemical reduction. Furthermore, the gamma radiation reduces the aggregations of graphene nanosheets owing to its mild irradiation reduction rate [16]. In this study, we reported a salinization of GO by gamma ray irradiation. GO were functionalized by two different silane agents namely 3-aminopropyltriethoxysilane (APTES) and 3-glycidyloxypropyltrimethoxysilane (GPTES) (Figure 1) at different gamma radiation doses.

## 2. Results and Discussion 

### 2.1. Fourier Transform Infrared (FTIR)

**Figure 2:**
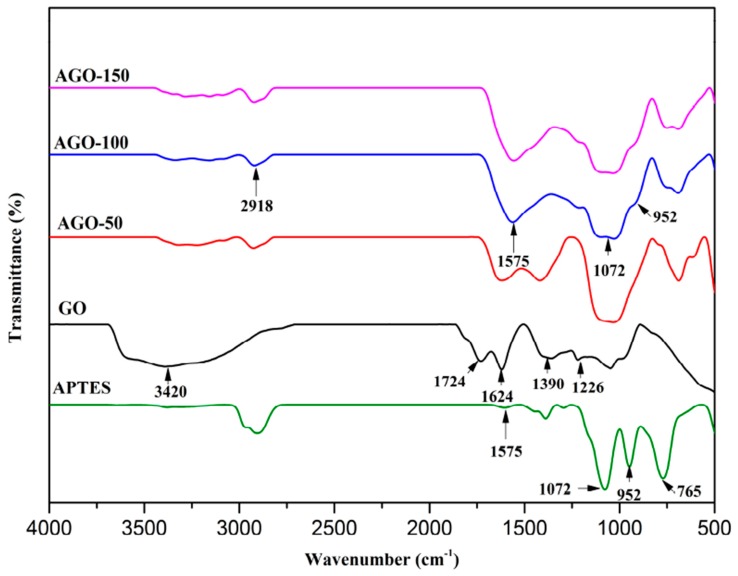
FTIR spectra of GO and 3-aminopropyltriethoxysilne functionalized-GO (AGO-50, AGO-100 and AGO-150).

**Figure 3:**
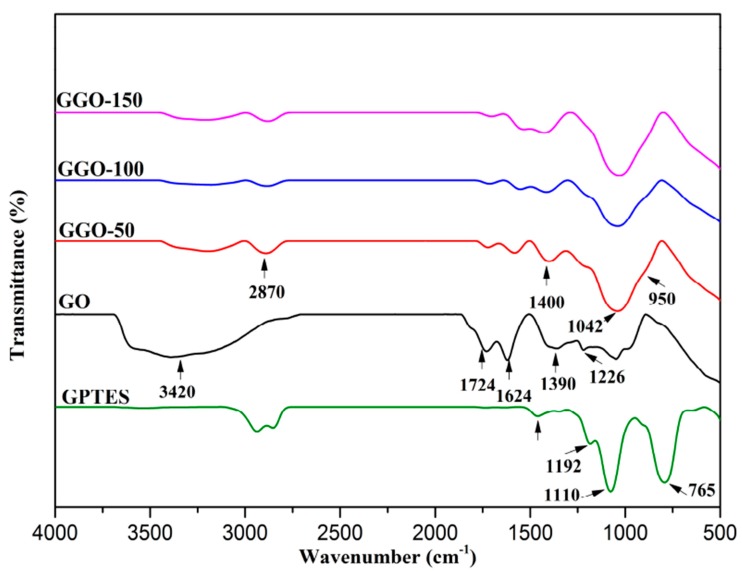
FTIR spectra of GO and 3-glycidyloxypropyltrimethoxysilane functionalized-GO (GGO-50, GGO-100 and GGO-150).

FTIR analysis is one of the direct evidences for GO functionalization as information about the presence of functional groups in the samples. Figure 2 and Figure 3 show the FTIR spectra for 3-aminopropyltriethoxysilne functionalized-GO (AGO) and 3-glycidyloxypropyltrimethoxysilane functionalized-GO (GGO), respectively. The characteristics peaks of APTES at 1574 to 1576 cm^−1^ is assigned to the deformation vibration of N-H bond, 1296 and 765 cm^−1^ assigned to Si-C vibration, and the 1165, 1072, and 962 cm^−1^ which were assigned to Si-O-C bond [17]. The peak at 1100 cm^−1^ is assigned to the stretching vibration of C-NH_2_ bond [18,19]. The characteristics features of GO includes the absorption band at 1624 cm^−1^ (C=C skeletal vibration), 1068 and 1227 cm^−1^ (C-O-C in epoxide group), 3420 cm^−1^ (C-OH stretching), 1390 and 1724 cm^−1^ (C=O stretching vibration of carbonyl groups), 2830 and 2917 cm^−1^ (symmetric and asymmetric bands). For AGO, a new characteristic band appears at 1072 and 952 cm^−1^ which is assigned to Si-O-C and Si-O-Si indicating the successful chemical functionalization since the characteristics peaks of GO which include C=O vibration and carboxylic groups at 1724 cm^−1^ and CH_2_ at 2926 cm^−1^ vibration bands were not observed in the functionalized-GO. These changes can be observed for all the functionalized samples at different radiation doses of AGO-50, AGO-100, and AGO-150.

Strong characteristic peaks of GPTES was observed at 1196 and 1100 cm^−1^ which corresponded to CH_2_ wagging vibrations of propyl chain and glycidoxy group, respectively [20]. For GGO, the band at 3420 cm^−1^ becomes weak and band at 2870 cm^−1^ appears corresponding to the stretching of -CH_2_ groups from alkyl chain assigned to silane mioties of silane as shown in Figure 3. The appearance of bands at 1042 and 950 cm^−1^ which are assigned to Si-O-C and Si-O-Si bonds, respectively, indicating the successful chemical functionalization. 

### 2.2. X-ray Diffraction (XRD) Analysis

**Figure 4:**
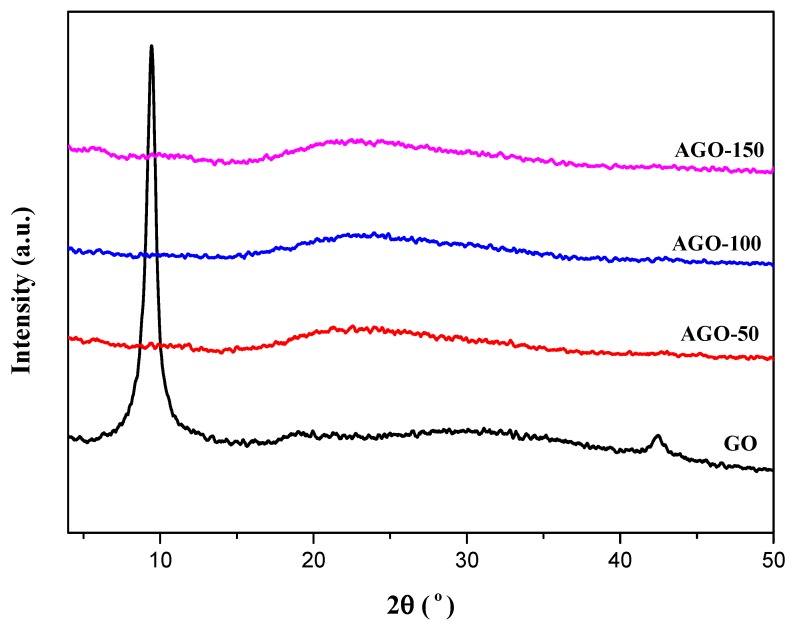
XRD spectra of GO and 3-aminopropyltriethoxysilne functionalized-GO (AGO-50, AGO-100, and AGO-150).

The XRD diffractograms of GO and AGO are shown in Figure 4. The GO shows a sharp peak at 2Ɵ = 9.8 corresponding to the (001) plane with an interlayer spacing (d-spacing) of 0.90 nm indicating a highly ordered structure. This large d-spacing of GO results from the presence of large amount of oxygen functional groups on the surface of GO. The XRD pattern also shows narrow (100) peak with low intensity that corresponds to graphitic plane with long range order [21]. After functionalization of GO with APTES, the XRD characteristic peak of the GO disappear due to the intercalation of silane moieties on the surface of GO which leads to partial reduction and complete exfoliation of GO which in turn leads to prevention of aggregation [22]. The disappearance of (001) peak in the case of AGO-50, AGO-100 and AGO-150 indicates the loss of order perpendicular to the GO layer and complete loss of planarity of functionalized-GO due to loss of (100) reflection. Similarly, the emergence of broad peak around 22.19° suggest the removal of oxygen functional groups and dominant effect of silane [23].

**Figure 5:**
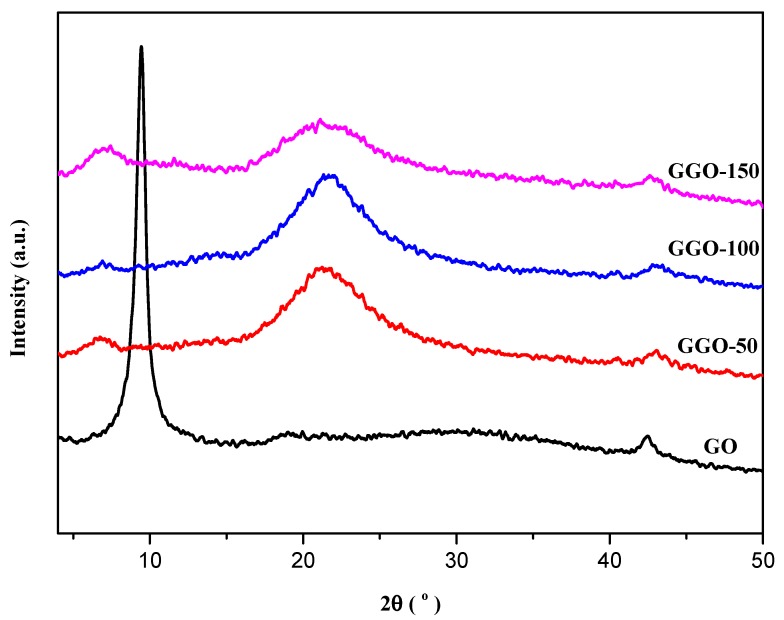
XRD spectra of GO and 3-glycidyloxypropyltrimethoxysilane functionalized-GO (GGO-50, GGO-100, and GGO-150).

The diffractograms of GGO are shown in Figure 5. The appearance of broad band with low intensity cantered at 21.4° reveals the dominance of silane moieties and the removal of oxygen containing groups on the GO surface. The emergence of a new hump at 2Ɵ = 7.2° in all of the GO functionalized with 3-glycidyloxypropyltrimethoxysilane at GGO-50, GGO-100, and GGO-150 with varying intensity revealed covalent functionalization of the GO with silane moieties. The XRD patterns of functionalized-GO—i.e., AGO’s and GGO’s—are moderately similar except for the fact that for GGO’s pattern has higher intensity compared to AGO’s. This is attributed to the extent of reduction by 3-glycidyloxypropyltrimethoxysilane that also have a broad peck at 21.3° which subsequently enlarge the interlayer distance (002) [22].

### 2.3. Field Emission Scanning Electron Microscopy (FE-SEM)

**Figure 6:**
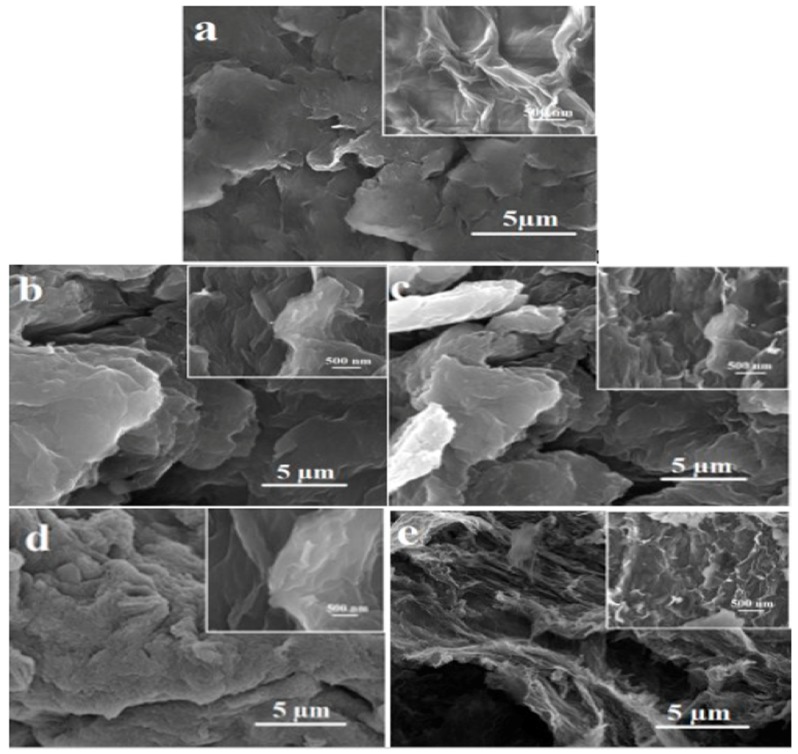
FE-SEM micrographs of (**a**) GO, (**b**) AGO-100, (**c**) GGO-100, (**d**) AGO-150, and (**e**) GGO-150.

Surface morphologies of pristine GO and functionalized-GO were observed using FE-SEM. Pristine GO has smooth surface with layered structure at the edges [1] (Figure 6a). On the other hand, Figure 6b AGO-100, Figure 6c GGO-100 and Figure 6d AGO-150 and Figure 6e GGO-150 samples exhibit rough surface with wrinkle morphology and the inserted magnified image on all the four samples shows several thin sheets with puffy structures [22], which signify the present of silane moieties that act as a spacer between the GO sheets and hence preventing restacking [24,25]. The distinction in morphology observed between GO and the functionalized-GO results from covalent bonding and condensation reactions between silanes molecules and GO sheets which subsequently hinders agglomeration [26].

### 2.4. Raman Spectroscopy

**Figure 7:**
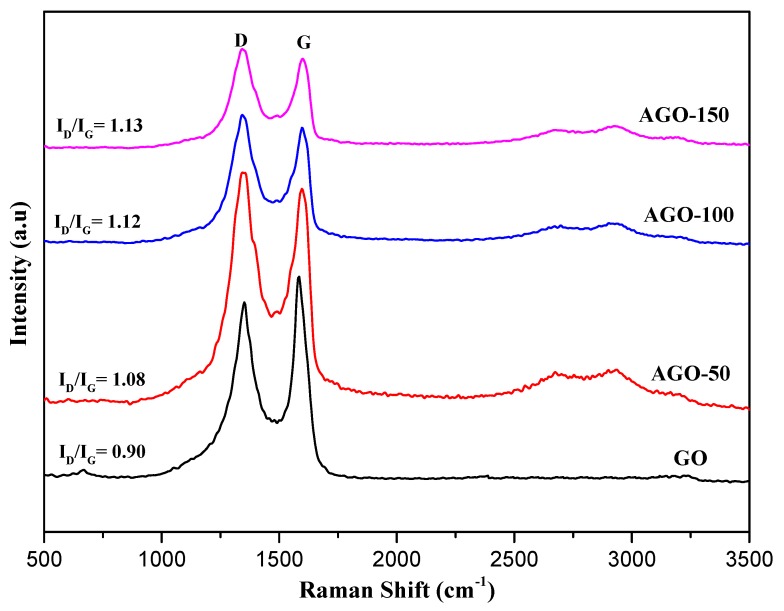
Raman spectra of GO and 3-aminopropyltriethoxysilane functionalized-GO (AGO-50, AGO-100 and AGO-150).

**Figure 8:**
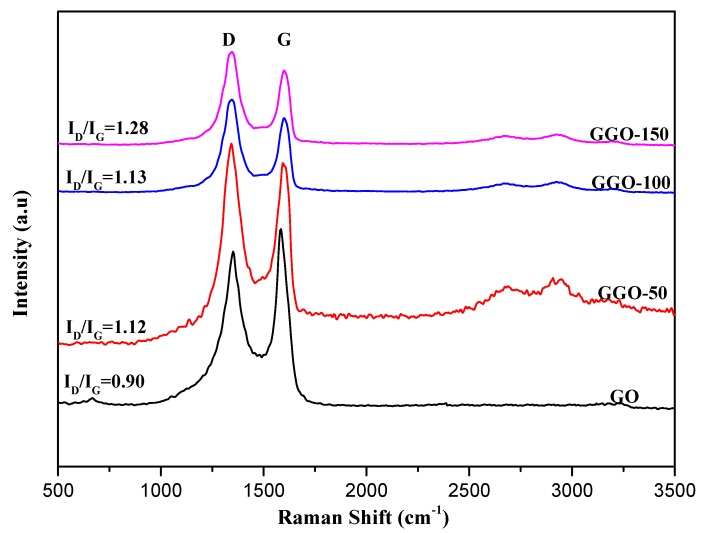
Raman spectra of GO and 3-glycidyloxypropyltrimethoxysilane functionalized-GO (GGO-50, GGO-100, and GGO-150).

The Raman spectroscopy is a powerful tool used to investigate the structure of a graphene-based material. The Raman spectra of GO and functionalized-GO are shown in Figure 7 and Figure 8. GO exhibit two strong characteristics bands at 1583 cm^−1^ (G band) and 1345 cm^−1^ (D band). The D and G bands are attributed to the structural defect (disorder-induced mode) and first order scattering from the E2g phenom of sp^2^ carbon bonding, respectively. The ratio of the intensities between the D and G bands (I_D_/I_G_) depends on the level of disorder; i.e., sp^3^ defects within the sp^2^ hybridized graphene in the functionalized-GO [6]. The I_D_/I_G_ ratios for GO, AGO-50, AGO-100, and AGO-150 were 0.90, 1.08, 1.12, and 1.13, respectively as shown in Table 1. The increase in I_D_/I_C_ ratio clearly demonstrate the defect in graphene sheet caused by the silane grafting that might leads to considerable changes in the physic-chemical, structural and electrical properties. This result shows that the functionalization of GO with APTES alters the structure of GO through the formation of more sp^3^ carbon within the sp^2^ carbon network of graphene, resulting in higher of I_D_/I_G_ in AGO [27]. The forward shifting of G band position also confirm the assimilation of silane moieties in the GO framework implying better exfoliation of graphene layer [3].

Similarly, the I_D_/I_G_ values for GGO-50, GGO-100, and GGO-150 are 1.12, 1.13, and 1.28, respectively. The observed ratio of GO functionalized with GPTES are almost similar but slightly higher than that functionalized with APTES, this could be probably due to the high extend of grafting by APTES. 

### 2.5. Thermogravimetric (TG) Analysis

**Figure 9:**
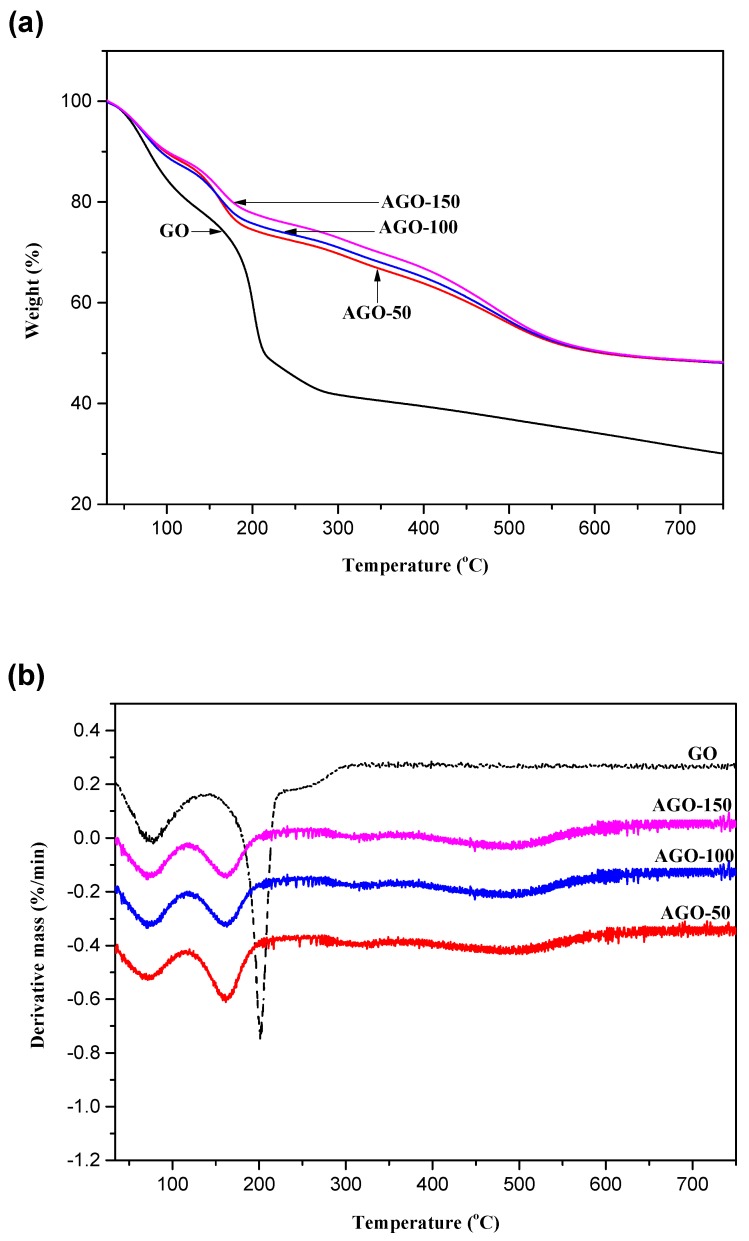
(**a**) TG and (**b**) DTG thermograms of GO, AGO-50, AGO-100, and AGO-150.

TG analysis was used to investigate the thermal stability of the sample and to further confirm the functionalization of GO with silane moities. The TG and derivative thermogravimetric (DTG) thermograms of AGOs are shown in Figure 9. Upon heating, GO loses ~18% weight at temperature between 30–120 °C due to the removal of water molecules that adsorbed onto the hydrophilic GO surface. As the temperature increases from 150–230 °C GO exhibit decomposition behavior (39% weight loss) which is attributed to the labile oxygen containing functional groups. Another significant weight drop occurs around 300 °C where GO loses about 68% weight which is due to pyrolysis of the carbon skeleton which is referred to as end degradation temperature. The TGA thermograph depicted in Figure 9a represent the thermal behavior of APTES grafted GO (AGO-50, AGO-100, and AGO-150). Four steps degradation mechanism are shown for all the functionalized-GO at various temperature regions. These regions are 0–120 °C corresponding to region where water molecules are lost, 120–300 °C where unreacted silane is adsorbed onto the surface of the GO are lost, 300–650 °C corresponding to region of thermal decomposition of grafted silane and at 650 °C and above where pyrolysis of the carbon skeletal took place. As compared to GO which is represented by three degradation steps in the DTG shown in Figure 9b. In the case of functionalized-GO, the second step degradation (i.e., between 120–300 °C) involves the removal of physically adsorbed silane molecules while the degradation step between 300–650 °C can be ascribed to the breakage of APTES molecules grafted to the GO surface. This thermal behavior is well pointed out in many studies conducted on graphene and carbon nanotube [28,29]. The end temperature of degradation i.e., pyrolysis of the carbon skeletal significantly shifted to a higher temperature as a result of silane grafting thereby enhancing thermal stability [30]. Table 2 highlights the approximate percentage weight loss of the samples at various temperature regions.

**Figure 10:**
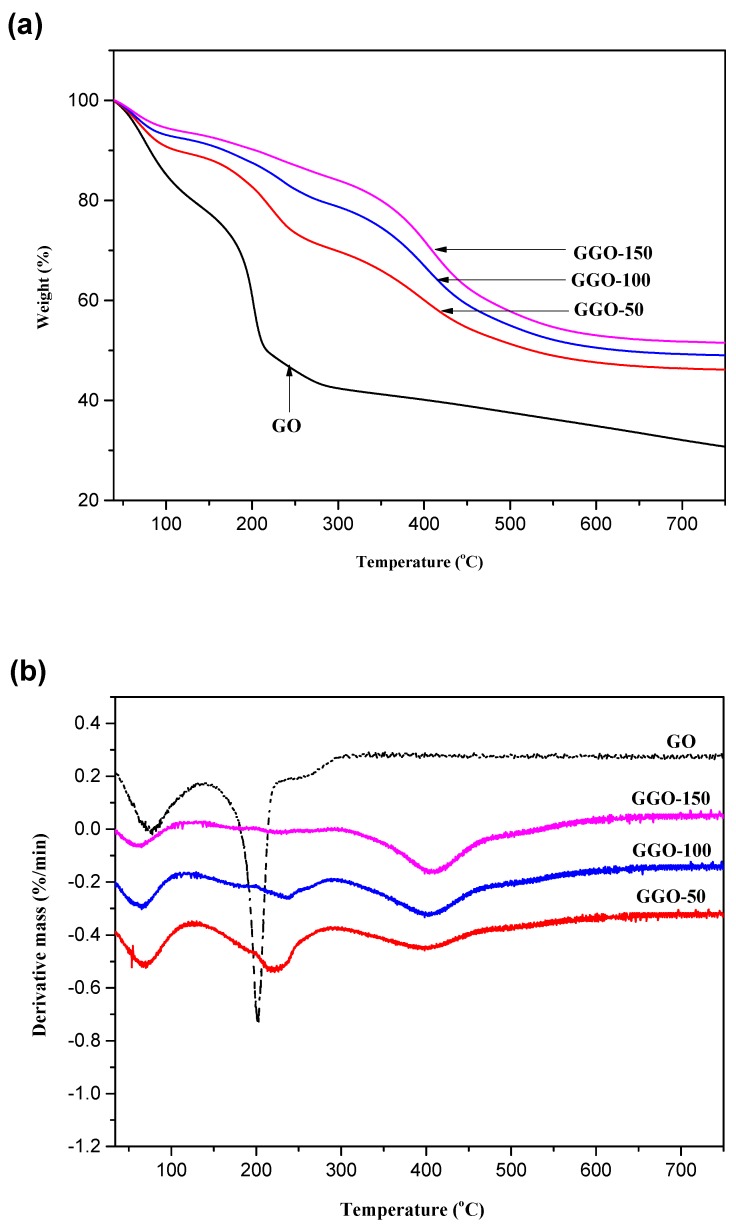
(**a**) TG and (**b**) DTG thermograms of GO, GGO-50, GGO-100, and GGO-150.

On the other hand, the weight loss regions for the GGO can be observed in the thermograms depicted in Figure 10, which are at 0–120 °C, 140–320 °C, and 350–500 °C temperature regions. Similarly, these regions also correspond water evaporation, loss of unreacted silanes adsorbed at the surface of GO and oxidative thermal decomposition of grafted silanes. GGO-150 shows the lowest weight loss of 5% at the 0–120 °C temperature region with enhance thermal stability than other functionalized-GO.

As stated earlier, the weight loss of the silane functionalized-GO comes from three contributions. The first contribution was the dehydration and dehydroxylation which occurs during heating process in the TGA measurement [31]. In the temperature range between 30–120 °C, the weight loss was mainly due to the elimination of water molecules adsorbed on the GO surface [32]. As the temperature rises above 120 °C all the molecular water was removed and any other weight loss comes from APTES and GPTES molecules respectively. The second contribution is as a result of physically adsorbed or decomposed APTES and GPTES which can be removed by washing with ethanol. However, complete removal is hardly achieved, therefore any possible remains still gives a contribution to the weight loss during TGA measurement. The boiling point of APTES and GPTES is 217 °C and 120 °C, respectively; it is therefore assumed all physically adsorbed APTES and GPTES were completely removed before 300 °C. The third contribution comes from the decomposition of chemically bonded APTES and GPTES. From the deferential curves, it can be seen that the main decomposition occurs around 500 °C which is agreement with previous study [33] that the thermal decomposition of grafted silane took place above 450 °C and the C-Si bond started breaking up at 450–510 °C when APTES was heated in a nitrogen gas atmosphere.

## 3. Materials and Methods

### 3.1. Materials

Graphite powder (<20 µm) was obtained from Sigma Aldrich (St. Louis, MO, USA), sulphuric acid (H_2_SO_4_, 98%) and hydrogen peroxide (H_2_O_2_, 30%) were obtained from Baker Analyzed (Selangor, Malaysia) and used as received. Potassium permanganate (KMnO_4_, 99.9%) and hydrochloric acid (HCl, 36%) were purchased from R&M chemicals (Selangor, Malaysia) 3-aminopropyltriethoxysilane and 3-glycidyloxypropyltrimethoxysilane were obtained from Sigma Aldrich. All other chemicals were of analytical grade and used as received without further purification. Distilled water was used for the washings.

### 3.2. Synthesis of Graphene Oxide

GO was synthesized from graphite using a simplified Hummers’ method. 3g of graphite was first oxidized by reacting them with concentrated H_2_SO_4_ (400 mL) before adding KMnO_4_ (18 g). The reaction was allowed to go on for 60 h to fully oxidize graphite to graphite oxide. 10% *v*/*v* H_2_O_2_ solution was added to destroy excess KMnO_4_ and terminate the oxidation process. The GO formed was washed several times with 1 M of aqueous HCl solution to remove SO_4_^2^^−^ ions. Then, the mixture was washed repeatedly with distilled water to remove Cl^−^ ions in the mixture. The washing process was carried out using a simple decantation of the supernatant by centrifugation technique. The final product was freeze dried.

### 3.3. Functionalization of Graphene Oxide

A silane solution is prepared and added to a solution of 50 mL of 2.5 gL^−1^ of as-prepared GO and sonicated for 30 min. The mixture is stirred for 30 min before irradiation. The ^60^Co gamma ray source with an activity of 0.37 MCi (Mega Curie), (MDS Nordion Inc, Ottawa, ON, Canada) was used at room temperature atmosphere with the total doses of 50 kGy, 100 kGy and 150 kGy. After irradiation treatment, the mixtures were centrifuge at 4000 rpm for 2 min. After that the supernatant was removed. The residues denoted as AGO-50, AGO-100, AGO-150 for 3-aminopropyltriethoxysilane functionalized-GO and GGO-50, GGO-100, GGO-150 for 3-glycidyloxypropyltrimethoxysilane functionalized-GO residue was washed five times with mixture of (60:40 *w*/*w*) deionize water and ethanol to remove unreated silane. The final product was freeze dried. 

### 3.4. Characterizations

Fourier transform infrared (FT-IR) spectra were recorded using a spectrum 100 Perkin Elmer (Waltham, MA, USA). The FT-IR spectra of the samples were recorded in the range of the wavelength 280 to 4000 cm^−1^ at 25 °C. A Mettler Toledo 1 HT (Columbus, OH, USA) was used for thermogravimetric analysis (TGA) of the samples. About 10–15 mg of each sample was used for the analysis. The samples were heated from 35 °C to 800 °C at the heating rate of 10 °C/min. The analysis was carried out at nitrogen flow rate of 20 mL/min. The weight loss temperature function graph was plotted. Shimadzu XRD-6000 X-ray diffractometer (Tokyo, Japan) was used to determine the interlaying spacing of the GO sheets before and after functionalization. Data were collected within the range of scattering angles (2θ) of 2° to 50° at the rate of 2°/min. Raman spectroscopy was used to evaluate the microstructure using Alpha300R Laser Raman spectrophotometer (WItec, Ulm, Germany). The Raman shift was recorded at 500–4000 cm^−1^ wavelength region. The surface morphology was characterized by FE-SEM using FEI Nova NanoSEM 230 (FEI, Hillsboro, OR, USA).

## 4. Conclusions

In this study, 3-aminopropyltriethoxysilne (APTES) and 3-glycidyloxypropyltrimethoxysilane (GPTES) were successfully grafted onto GO via gamma-ray irradiation. The changes in structure and morphology due to radiation induced functionalization and partial reduction are proven by means of FT-IR, XRD, FE-SEM, Raman, and TG analyses. Beside the elimination of hydroxyl and epoxide functional groups, some alkyl groups are attached onto functionalized-GO due to recombination of radicals. This method is a promising way to functionalize and partially reduce GO at an even lower radiation dose of 50 kGy. Silane functionalized-GO has great potential for use as a hydrophobic material in industry such as in corrosion prevention application.

## Figures and Tables

**Table 1:** Summary of the Raman Shift and I_D_/I_G_ for the samples

Samples	Raman Shift of D Band (cm^−1^)	Raman Shift of D Band (cm^−1^)	I_D_/I_G_
GO	1345.77	1583.33	0.90
AGO-50	1345.77	1589.55	1.08
AGO-100	1345.77	1594.52	1.12
AGO-150	1345.77	1599.50	1.13
GGO-50	1345.77	1597.01	1.12
GGO-100	1345.77	1597.01	1.13
GGO-150	1345.77	1601.99	1.28

**Table 2:** Percentage weight loss of GO and functionalized-GOs at various temperature regions

Samples	Weight Loss (%) 30–120 °C	Weight Loss (%) 120–300 °C	Weight Loss (%) 300–650 °C
GO	18.7	39.7	0
AGO-50	12.0	18.3	20.5
AGO-100	12.8	16.2	21.6
AGO-150	11.8	15.6	23.4
GGO-50	11.0	19.8	23.0
GGO-100	8.4	13.7	29.0
GGO-150	7.0	9.8	31.8
